# Guillain-Barre Syndrome After COVID-19 Vaccination: A Secondary Analysis of Domestic Safety Data by the Japanese Government

**DOI:** 10.7759/cureus.30905

**Published:** 2022-10-31

**Authors:** Yudai Kaneda, Takanao Hashimoto, Uiri Kaneda, Yuka Higuchi, Jun Murakami, Masanari Inada, Yuki Senoo, Takeshi Fujieda, Yuki Murata, Tetsuya Tanimoto

**Affiliations:** 1 School of Medicine, Hokkaido University, Sapporo, JPN; 2 Department of Pharmacy, Kenkodo Pharmacy, Osaki, JPN; 3 Faculty of Foreign Languages, Dokkyo University, Soka, JPN; 4 School of Medicine, Kyushu University, Fukuoka, JPN; 5 Department of Transfusion Medicine and Cell Therapy, Toyama University Hospital, Toyama, JPN; 6 Department of Rehabilitation Medicine, Yokohama City University Hospital, Yokohama, JPN; 7 Department of Internal Medicine, Medical Governance Research Institute, Tokyo, JPN; 8 Department of Internal Medicine, Kitaibaraki Municipal Medical Center, Kitaibaraki, JPN; 9 Department of Paediatrics, Tokyo Metropolitan Children’s Medical Center, Tokyo, JPN; 10 Department of Internal Medicine, Navitas Clinic, Tokyo, JPN

**Keywords:** japan, adverse reactions, mrna vaccines, covid-19, guillan-barre syndrome

## Abstract

Purpose

The purpose of this study was to figure out the risk of Guillain-Barre syndrome (GBS) after coronavirus disease 2019 (COVID-19) vaccination, which has been reported as a rare adverse reaction.

Methods

Elucidating the characteristics, we performed a secondary analysis of the cases from February 2020 through January 2022, based on the publicly available spontaneous adverse reaction reports in Japan.

Results

We identified 115 cases, and all were after messenger RNA (mRNA) vaccination. Of all the cases, 69 (60.0%) were female and 44 (38.2%) were older than 65 years old. Severe GBS was reported by 38 males (median age 61.5 years) and 51 females (median age 55 years). The median interval from vaccination to the onset of symptoms was eight days for males and four days for females. Sequelae were reported in 18 patients (7 males, median age 81 years; 11 females, median age 51 years), 11 of whom were older than 65 years old. The estimated incidence was about 0.0001% (0.000058% for the Pfizer vaccine and about 0.000046% for the Moderna vaccine, respectively).

Conclusions

Spontaneous reports would have various biases, the incidence of GBS after mRNA vaccination was as low as in other existing vaccination programs, and it is important not to interpret that risk expansively.

## Introduction

As a countermeasure against the coronavirus disease 2019 (COVID-19) pandemic, vaccination has been administered worldwide, and by March 2022, more than 55% of the world's population has already completed two doses of vaccination regardless of the type of product [1]. Moreover, booster vaccinations are widely recommended against the prevalent Omicron variant, and third and fourth doses of vaccination are being promoted worldwide [2].

However, various adverse reactions after vaccination have been reported such as fever, joint pains, and anaphylaxis [3,4]. Among them, Guillain-Barre Syndrome (GBS) is also one of the rare adverse reactions after vaccination. GBS has been reported after the use of conventional vaccines, such as inactivated influenza vaccines [5], and cases of GBS have been reported worldwide even with the newly developed COVID-19 vaccines [6-13]. The pathogenesis of the disease is assumed to be associated with vaccine-induced production of type 1 interferon, which reduces resistance to myelin sheath antigens, but the details have not yet been identified [14].

In Japan, more than 230 million doses of vaccine, including boosters, have been provided as of March 2022, where three vaccine products were approved by Japan’s regulatory authority, namely, two mRNA vaccines manufactured by Pfizer and Moderna, and the ChAdOx1 vaccine manufactured by AstraZeneca [15]. In addition, on April 19, 2022, a recombinant protein vaccine developed by Novavax was newly approved by the regulatory authorities as the COVID-19 vaccine, and its vaccination will begin in late May 2022 at the earliest [16].

Although the Ministry of Health, Labor, and Welfare (MHLW) is disclosing a list of suspected adverse reactions spontaneously reported by each medical institution, details of GBS in Japan have not been reported. With official, publicly available data of spontaneous reports from the MHLW website, we conducted a secondary analysis focused on GBS after COVID-19 vaccination in Japan.

## Materials and methods

The Pfizer vaccine was first approved by Japan’s regulatory authority on February 14, 2021, and vaccination began on February 17, 2021, in the country [17]. Then, the Moderna and AstraZeneca vaccines were approved on May 21, 2021, and vaccination with the Moderna vaccine began on the twenty-fourth of the same month [17]. The Pfizer and Moderna vaccines are administered at intervals of three and four weeks, respectively, for those 12 years of age and older [18]. As for the AstraZeneca vaccine, as of August 3 of the same year, in accordance with the Immunization Law, vaccinations were to be given at intervals of 4 to 12 weeks, generally for persons 40 years of age and older [18]. In Japan, the Moderna vaccine is used for workplace vaccinations, so younger people tend to receive the Moderna vaccine, and older people tend to receive the Pfizer vaccine, which is distributed at medical institutions [19].

We surveyed the information disclosed as of February 18, 2022, on the MHLW website regarding adverse reactions spontaneously reported by Japanese medical institutions from February 17, 2021 (the start of the COVID-19 vaccination program in Japan) to January 23, 2022 [20]. For the diagnosis of GBS, each medical institution spontaneously reported possible cases based on their own judgment as well. We collected information from these reports on the age, sex, vaccine type, number of days in the interval between vaccination and onset of GBS symptoms, causal relationship judgment by the attending physicians, outcome details, and severity of GBS. In addition, we calculated the frequency of GBS incidence by obtaining the usage frequency of each vaccine and the number of people who had completed at least one vaccination until the last day of the study period from publicly available data from the website of the Japanese Cabinet Office [19].

Statistical analysis was not performed due to the small number of subjects with GBS. Ethical considerations were not applied to this study as we used only publicly available data.

## Results

During the study period, a total of 116 patients with GBS were reported. Among them, we judged that there was a duplication of data about the same person and excluded one of them. Of the remaining 115 patients as shown in Table 1, the median age was 49 years (range 13 to 89 years), 44 patients (38.2%) were older than 65 years, and 5 patients (4.3%) were younger than 20 years. Sixty-nine patients (60.0%) were female and 46 (40%) were male.

**Table 1:** Characteristics of patients who developed Guillain-Barre syndrome in Japan ※We judged that there was a duplication of data about the same person and excluded one of them.

Variables	All (N=115)
Age (Median, Range)	49 (13 - 89)
Interval (Median, Range)	6 (0 – 76)
Sex	
Male	46
Female	69
Type of vaccination	
Pfizer	100
Moderna	15
Severity	
Severe	89
Not severe	26
Correlation judged by attending physicians	
Yes	56
Not clear	54
Unknown	5
Outcomes	
Recovery	7
Mild recovery	41
Sequelae	18
Death	1
Unknown	21
Unrecovered	27

**Figure 1:**
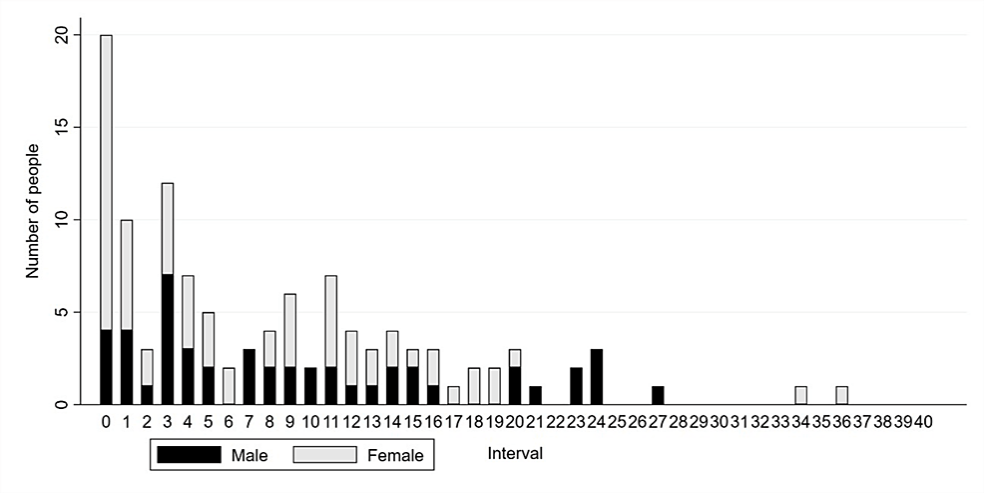
The relationship between the interval from vaccination to Guillain-Barre syndrome onset and the number of men and women ※A woman who developed Guillain-Barre syndrome 76 days after her vaccination is not shown in the figure.

Figure 1 shows the relationship between the interval from vaccination to the onset of GBS and the number of men and women. The median interval from vaccination to symptom development was six days (range 0 to 76 days) as a whole, eight days (range 0 to 27 days) for males, and four days (range 0 to 76 days) for females. The association between vaccination and the development of GBS was judged to be positive in 56 patients (48.7%) with a median interval of four days (range 0 to 23 days), to be unknown in 54 patients (47.0%) with a median of 12 days (range 0 to 76 days). The remaining five cases did not have this information. By age, teens had the longest median interval of 20 days (range 16 to 27 days), followed by the 70s with 10 days (range 0 to 24 days). The shortest median intervals were three days in the 30s and 40s (range 0 to 15 and 0 to 20 days, respectively).

By the type of vaccine, 100 patients (87.0%) were after the Pfizer vaccine, 15 patients (13.0%) were after the Moderna vaccine, and none were after the AstraZeneca vaccine. For the Pfizer vaccine, the median age was 53.5 years (range 13 to 89), and the median interval was five days (range 0 to 76 days). For the Moderna vaccine, the median age was 43 years (range 26 to 64), and the median interval was nine days (range 0 to 20 days). Severe GBS was observed in 89 patients (77.4%; nine from Moderna and 80 from Pfizer) with a median age of 57 years (range 13 to 89). Of these, 38 were male (median age 61.5 years) and 51 were female (median age 55 years). Non-severe GBS was observed in 26 patients with a median age of 43 years (8 males, median age 51;18 females, median age 41).

Concerning outcomes, a recovery trend was reported in a total of 48 patients (7 as recovery and 41 as mild recovery). Of the seven recovered patients, two were male and five were female, and their average age was 40 years old. Sequelae were reported in 18 patients. Moreover, one case of death of a 71-year-old man and 27 cases of non-recovery were reported. No reports were obtained in 21 patients. The median age of the 18 patients with sequelae was 68.5 years (7 males, 81 years; 11 females, 51 years), and 11 patients were older than 65 years old.

In terms of the incidence of GBS, as of the last day of the study period, approximately 105 million people (80.4% of the Japanese population) had received at least one dose of vaccination, translating into about 0.0001%. In terms of the number of vaccinations, the estimated number of vaccinations up to January 23, 2022, was 170,977,414 for the Pfizer vaccine and 32,557,064 for the Moderna vaccine. Calculating with these numbers, the incidence of GBS after vaccination is 0.000058% for the Pfizer vaccine and 0.000046% for the Moderna vaccine.

## Discussion

This is the first study to report a detailed examination of GBS after COVID-19 vaccination in the Japanese using publicly available spontaneous reports. The mRNA COVID-19 vaccines are mainly used in Japan, and all cases of GBS reported after vaccination were after those vaccines. Patients were mainly elderly, and females were the majority. The interval from vaccination to disease onset was shorter for females, and it was shortest for patients in their 30s and 40s.

The frequency of GBS in Japanese after COVID-19 vaccination was about 0.0001%. Additionally, the incidence of GBS after each vaccination was about 0.000058% for Pfizer and 0.000046% for Moderna, suggesting that the Pfizer vaccine offers more risk of developing GBS after vaccination. On the other hand, in the United States (US), the incidence of GBS after mRNA vaccination against COVID-19 was reported to be more than 0.001% [21], translating the incidence was about one-tenth among the Japanese compared to the Americans. There should be some biases, for example, in terms of reporting systems; in the US, the adverse reaction reporting site Vaccine Adverse Event Reporting System (VAERS) has settings that allow both healthcare professionals and patients to report adverse reactions to vaccines through a smartphone portal called "V-safe," and the reporting by healthcare professionals is at least mandatory [22]. On the other hand, in Japan, healthcare professionals who suspect an adverse reaction are required to follow complicated procedures such as obtaining lot numbers of vaccines and then reporting to the government office, which may lead to under-reporting of spontaneous reports [20], Various biases cannot be excluded, but the risk of developing GBS after mRNA COVID-19 vaccination was found to be as low as previous studies on other vaccines [5].

Among the patients, females were predominant; those younger than 20 years old were rare while those older than 65 years old were more than one-third. In general, GBS tends to develop in the elderly, and the risk in males is reported to be about 1.8 times higher than that in females [23,24]. In terms of age, our finding was consistent. In addition, anaphylaxis after mRNA COVID-19 vaccination was more frequent in women in reports from both Japan and the US [25,26]. Thus, the sexual difference in adverse reactions after mRNA vaccines warrant further investigation [27].

For those with a presumed causal relationship, the median interval for GBS development was four days compared with 12 days for those with an unknown relationship. There may be a strong reporting bias here because shorter intervals would be more easily judged to be relevant. Although the Japanese government has stated that "the Adverse Reaction Study Committee is closely monitoring this issue for future evaluation," there is no defined follow-up management system for adverse reactions after vaccination except for anaphylaxis [28]. On the other hand, the research team in the US indicates that the incidence of general adverse reactions after COVID-19 vaccination, in general, is not significantly higher in the first three weeks than in the following three weeks [29]. Therefore, it is important to establish a follow-up management system that can provide healthcare for not only short-term but also long-term adverse reactions after vaccination. In particular, since the median interval was relatively longer for teenagers and the elderly in their 70s (20 and 10 days, respectively), more careful, long-term post-vaccination adverse reactions management should be established.

In this study, 89 were severe, but this may be because severe patients were more likely to be reported in the spontaneous reporting system. Since the Guillain-Barre Fisher Syndrome Guidelines 2013 define treatment as Grade 2 or higher on the Hughes Functional Grade [30], it is likely that patients equivalent to Grade 1 were not sufficiently reported. In addition, the median age of those who were seriously affected was older than those who were not, again implying that older people tend to be more severely affected.

As for outcomes, of 18 reported cases of sequelae, women were much more likely than men to have sequelae, even at younger ages. In addition, the majority of patients with sequelae were over the age of 65, suggesting that they are a high-risk group. Although many patients showed a recovery trend, it is important to continue the follow-up since it has been reported that about 10% of GBS patients show treatment-related fluctuation in the long run [31].

There are several limitations to this study. First, since this study was based on a public database disclosed by the MHLW, we could not obtain the patients’ underlying diseases and conditions or whether the development of GBS was after the first or second vaccination. Second, for symptoms other than anaphylaxis, the period of observation for adverse reactions is completely dependent on each medical institution’s discretion, and the details of the sequelae would not be determined if the period of observation is limited. In addition, the severity was based on reports from each physician or medical institution, and there are no standardized diagnostic criteria; we should be careful with their interpretation. Third, in Japan, the incidence of GBS is generally estimated to be about 1.15/100, 000 people/year, and our study may include some natural cases [24].

## Conclusions

In conclusion, this is the first report of accumulated spontaneous reports on Japanese GBS patients after mRNA COVID-19 vaccines. The incidence of GBS after mRNA vaccination was as low as in other existing vaccination programs. Hence, it is important not to interpret that risk expansively.
